# Rampant Nuclear Insertion of mtDNA across Diverse Lineages within Orthoptera (Insecta)

**DOI:** 10.1371/journal.pone.0110508

**Published:** 2014-10-21

**Authors:** Hojun Song, Matthew J. Moulton, Michael F. Whiting

**Affiliations:** 1 Department of Biology, University of Central Florida, Orlando, Florida, United States of America; 2 Department of Human Genetics, University of Utah, Salt Lake City, Utah, United States of America; 3 Department of Biology and M. L. Bean Museum, Brigham Young University, Provo, Utah, United States of America; University of Innsbruck, Austria

## Abstract

Nuclear mitochondrial pseudogenes (numts) are non-functional fragments of mtDNA inserted into the nuclear genome. Numts are prevalent across eukaryotes and a positive correlation is known to exist between the number of numts and the genome size. Most numt surveys have relied on model organisms with fully sequenced nuclear genomes, but such analyses have limited utilities for making a generalization about the patterns of numt accumulation for any given clade. Among insects, the order Orthoptera is known to have the largest nuclear genome and it is also reported to include several species with a large number of numts. In this study, we use Orthoptera as a case study to document the diversity and abundance of numts by generating numts of three mitochondrial loci across 28 orthopteran families, representing the phylogenetic diversity of the order. We discover that numts are rampant in all lineages, but there is no discernable and consistent pattern of numt accumulation among different lineages. Likewise, we do not find any evidence that a certain mitochondrial gene is more prone to nuclear insertion than others. We also find that numt insertion must have occurred continuously and frequently throughout the diversification of Orthoptera. Although most numts are the result of recent nuclear insertion, we find evidence of very ancient numt insertion shared by highly divergent families dating back to the Jurassic period. Finally, we discuss several factors contributing to the extreme prevalence of numts in Orthoptera and highlight the importance of exploring the utility of numts in evolutionary studies.

## Introduction

It has been twenty years since the coining of the term “numts” to refer to nuclear mitochondrial pseudogenes [1], which are non-functional fragments of mtDNA inserted into the nucleus [2]. Initially considered abnormal and rare [3], [4], numts have since been reported from many divergent lineages of eukaryotes [2], [5], [6] and it is predicted that as more genomes are sequenced more numts will be discovered [5]. It has been well documented that mtDNA frequently escapes to the nucleus [7]–[10], and these mitochondrial fragments can be inserted into the chromosome during the repair of double-strand breaks in a mechanism known as non-homologous end-joining [11], [12]. Once inserted into the nuclear genome, numts become non-functional because of the differences in genetic code between mitochondrial and nuclear genomes [2], [8], [13]. Although there have been a number of promising advances made in the study of numts recently [11], [14]–[21], the exact mechanism of numt insertion and subsequent maintenance is still not fully understood [5].

Numts can be easily coamplified with mtDNA using conserved primers via conventional polymerase chain reactions [10], [22]–[28]. This is because numts have a relatively slower rate of substitution compared to mtDNA [13], [29], and the conserved primers would not only anneal to the desired mitochondrial sequences, but also to the corresponding sequences in the numts [23]. If the nuclear genome harbors a large number of coamplifiable numts, the resulting PCR products would contain both mtDNA and numts, which could result in ambiguous sequence reads [24], [28], [30]. In some cases, numts may be preferentially amplified to the mitochondrial sequences [25], [31]. Numerous earlier studies have highlighted the negative effects of numt coamplification in PCR-based research programs including population genetics [10], [25], [32], phylogenetics [26], [27], and DNA barcoding [23], [24], [30]. A number of studies have also proposed ways to reduce numt coamplification [2], [23], [24], [26], [30], [33]–[37], but currently there is no bulletproof and cost-effective method of completely eliminating numts. With incredibly rapid advances in sequencing technologies [38], generating complete mitochondrial genome sequences has become an easy feat [39] and thus the issue of numt coamplification may eventually become an irrelevant point in the near future.

However, numts are much more than simple nuisances to be avoided. They represent “molecular fossils” of extinct mtDNA lodged in the nucleus [13], [40], which has attracted a number of studies to explore their utilities in inferring evolutionary histories of various organisms including mammals, reptiles, and arthropods [14]–[18], [20], [41]–[43]. Because numts can remain intact in the nucleus for a long time [2], [5], [44], two taxa that share a common ancestor can potentially have numts that were inserted into the nuclear genome of the common ancestor [15], [18]. As such, a phylogenetic analysis of numts can reveal interesting patterns of past evolutionary events [14]–[18], [20], [45]. Nevertheless, there has not been any attempt to conduct a comprehensive survey of numts for a large and diverse clade. Instead, most surveys of numts have been based on available nuclear genomes that also have corresponding mitochondrial genomes [2], [5], [6], with little regard to taxon sampling. Although such surveys can reveal valuable insights, they are not currently practical for exploring the patterns of numt accumulation in non-model organisms.

In this study, we investigate the evolution of numts in the insect order Orthoptera, which includes familiar insects such as grasshoppers, katydids and crickets. It is the largest order within Polyneoptera including more than 26,000 extant species. Previous studies have suggested that there appears to be a positive correlation between the abundance of numts and the genome size [2], [5], [46], and Orthoptera has the largest known genome size among insects [46], [47]. As a comparison, the largest grasshopper genome is 16.56 Gb, which is 100 times larger than that of *Drosophila melanogaster* [48]. Thus, it is expected that the members of Orthoptera should harbor a large amount of numts, making it a particularly suitable group for studying numts. Several studies have already demonstrated the abundance of numts in different orthopteran species [4], [18], [23], [24], [49]–[52]. Furthermore, complete mitochondrial genomes have been sequenced for all major orthopteran lineages [53]–[56], making accurate numt identification and comparison feasible. Herein, we document the abundance of numts from 28 different families of Orthoptera, representing the entire phylogenetic diversity of the order. We use conventional PCR to coamplify numts and perform cloning reactions to sequence the resulting numts. By comparing them with the orthologous mtDNA, we identify and characterize numts and specifically address the following questions: (i) How widespread are numts across divergent lineages within Orthoptera?; (ii) Are there gene-specific and lineage-specific patterns?; and (iii) What are the patterns of numt accumulation in Orthoptera?

## Materials and Methods

### Taxon sampling

In order to survey the prevalence of numts across diverse lineages, we sampled 28 families representing 14 superfamilies across Orthoptera (Table 1). This taxon sampling included 19 families within the suborder Caelifera and nine families within Ensifera, therefore covering the phylogenetic diversity within the order (Table S1). In order to ensure the orthology of mitochondrial sequences used to compare with numts, we extracted appropriate sequences from the complete mitochondrial genomes of these 28 families as reference sequences. Of these, 17 have been published [53]–[55] and the remaining 11 were generated as part of senior author’s ongoing project on the phylogeny of Orthoptera, which are currently unpublished. For this study, we specifically targeted numts of three mitochondrial loci, cytochrome *c* oxidase subunit 1 (COI), cytochorome *c* oxidase subunit 2 (COII), and NADH dehydrogenase subunit 5 (ND5). For phylogenetic analyses, we used mitochondrial sequences of a mantid *Tamolanica tamolana* as an outgroup.

**Table 1:** A summary of numts of three mitochondrial genes (COI, COII, ND5) generated across 28 orthopteran families.

Taxonomic Information				COI				COII				ND5			
Suborder	Superfamily	Family	Species	total^a^	# ident.to orth.^b^	# uniquenumt^c^	# numtmutation^d^	total^a^	# ident.to orth.^b^	# uniquenumt^c^	# numtmutation^d^	total^a^	# ident. to orth.^b^	# unique numt^c^	# numtmutation^d^
Caelifera	Acridoidea	Acrididae	*Acrida* *willemsei*	47	35	12	1	43	25	18	0	43	33	10	1
Caelifera	Acridoidea	Lentulidae	*Lentula* *callani*	43	28	15	2	42	27	15	7	26	14	12	9
Caelifera	Acridoidea	Lithidiidae	*Lithiopsis* *carinatus*	42	18	24	8	46	27	19	9	24	8	16	9
Caelifera	Acridoidea	Pamphagidae	*Prionotropis* *hystrix*	46	24	22	3	40	19	20	16	42	31	11	4
Caelifera	Acridoidea	Pamphagodidae	*Hemicharilaus* *monomorphus*	46	31	15	1	16	13	3	0	30	3	27	27
Caelifera	Acridoidea	Pyrgacrididae	*Pyrgacris* *descampsi*	23	21	2	2	54	40	14	3	38	27	11	0
Caelifera	Acridoidea	Romaleidae	*Xyleus* *modestus*	45	31	13	3	47	30	14	0	45	27	18	2
Caelifera	Acridoidea	Tristiridae	*Tristira* *magellanica*	43	27	16	2	43	22	21	5	36	17	18	7
Caelifera	Eumastacoidea	Chorotypidae	*Chorotypus* *fenestratus*	36	27	9	1	16	15	1	0	45	41	4	3
Caelifera	Eumastacoidea	Eumastacidae	*Paramastax* *nigra*	40	16	23	5	9	2	7	7	45	27	18	3
Caelifera	Pneumoroidea	Pneumoridae	*Physemacris* *variolosa*	44	28	16	3	-	-	-	-	24	11	13	10
Caelifera	Proscopioidea	Proscopiidae	*Proscopia* sp.	44	0	8	0	27	0	8	8	42	31	11	7
Caelifera	Proscopioidea	Thericleidae	*Pseudothericles* *compressifrons*	32	20	12	3	24	13	11	1	41	30	10	0
Caelifera	Pyrgomorphoidea	Pyrgomorphidae	*Atractomorpha* *sinensis*	22	18	4	0	42	27	15	6	-	-	-	-
Caelifera	Tetrigoidea	Tetrigidae	*Trachytettix* *horridus*	61	2	32	9	-	-	-	-	17	12	5	4
Caelifera	Tridactyloidea	Cylindrachetidae	*Cylindraustralia* sp.	45	26	11	0	41	24	17	3	36	30	6	6
Caelifera	Tridactyloidea	Ripipterygidae	*Mirrhipipteryx* *andensis*	45	33	10	1	5	2	3	0	46	39	7	0
Caelifera	Tridactyloidea	Tridactylidae	*Ellipes minuta*	45	38	7	2	37	29	8	2	34	1	16	3
Caelifera	Trigonopterygoidea	Trigonopterygidae	*Trigonopteryx* *hopei*	37	22	15	5	6	2	4	3	32	16	16	9
Ensifera	Gryllacridoidea	Gryllacrididae	*Camptonotus* *carolinensis*	45	24	19	4	46	30	16	4	43	27	15	4
Ensifera	Grylloidea	Gryllotalpidae	*Gryllotalpa* *pluvialis*	33	28	3	0	-	-	-	-	-	-	-	-
Ensifera	Grylloidea	Myrmecophilidae	*Myrmecophila* *manni*	92	57	26	1	41	31	9	4	-	-	-	-
Ensifera	Hagloidea	Prophalangopsidae	*Cyphoderris* *monstrosa*	37	23	14	1	42	19	22	12	42	21	21	9
Ensifera	Schizodactyloidea	Schizodactylidae	*Comicus* *campestris*	46	38	8	0	9	8	1	0	44	33	11	0
Ensifera	Stenopelmatoidea	Anostostomatidae	*Henicus* *brevimucronatus*	18	14	4	1	13	12	1	0	-	-	-	-
Ensifera	Stenopelmatoidea	Rhaphidophoridae	*Troglophilus* *neglectus*	44	25	19	6	46	21	25	3	43	22	21	10
Ensifera	Stenopelmatoidea	Stenopelmatidae	*Stenopelmatus* *fuscus*	44	22	20	5	46	38	8	0	27	21	6	1
Ensifera	Tettigonioidea	Tettigoniidae	*Anabrus* *simplex*	68	27	41	12	36	17	9	0	29	16	13	0
			**Total**	**1213**	**703**	**420**	**81**	**817**	**493**	**289**	**93**	**874**	**538**	**316**	**128**

a “total” indicates the total number of clones sequenced from each PCR amplicon;

b “# ident. to orth.” indicates the number of cloned sequences that are identical to the orthologous mtDNA;

c “# unique numt” indicates the number of unique cloned sequences that are different from the orthologous mtDNA, thus representing numts;

d “# numt mutation” indicates the number of unique numts with characteristic in-frame stop codons or indels.

“-” indicates missing data.

### Numt generation

We followed the protocols described in Song et al. [24] and Moulton et al. [23] to generate numts. In short, we extracted genomic DNA from each species using Qiagen DNeasy kit from femur tissues. We have previously used this extraction protocol to successfully generate a large number of numts [18], [23], [24]. Because genomic DNA contains both mtDNA and nuclear DNA, a polymerase chain reaction (PCR) using conserved primers designed for mtDNA would co-amplify both orthologous mtDNA and numts. For PCR, we used a number of different primer pairs to generate the desired fragments and the details regarding the specific primers used for this study are listed in Table S2. Moulton et al. [23] showed that they were able to coamplify numts with both conserved primers and target-specific primers. Building upon their findings, we generally started with conserved primers for COI, COII, and ND5 for initial amplification, and tried more taxon-specific primers when the conserved primers did not yield any product. In all PCR for numt generation, we used Elongase Enzyme mix (Invitrogen Corporation, Carlsbad, CA, USA) in order to minimize and PCR and cloning errors, because of its high fidelity and low error rate (0.015% or 0.0987 bp per 658-bp COI Folmer region) [57]. Using TOPO TA Cloning Kit (Invitrogen Corporation), we cloned the resulting PCR amplicons and sequenced about 50 clones per reaction and characterized the resulting sequences. We used BigDye (version 3.1) chain terminating chemistry (Applied Biosystems Incorporated) to sequence the amplicons. The resulting sequences were proofread in Sequencher 4.8 (GeneCodes) and the sequences at each end that matched the primer sequences were removed. All resulting numts as well as mtDNA sequences have been deposited to GenBank with accession numbers KJ889444 - KJ890354.

### Sequence characterization

We followed the protocols described in Moulton et al. [23] to characterize the cloned sequences. In short, the resulting cloned sequences were first compared against the known mitochondrial sequences using MegaBLAST search in NCBI website. If the sequences did not return any similarity to insect mitochondrial genes, they were considered cloning errors and removed from further analyses. The remaining clones were categorized into those that were identical to the orthologs and those that were different from the orthologs, which we considered as numts. Then, these clones were compared against the appropriate orthologous sequence of a given species by aligning using MUSCLE (Edgar 2004) to infer the number of stop codons, indels and point mutations. Previous studies have shown that some numts do not contain stop codons and indels, and are seemingly functional [23]. Thus, we also calculated the sequence divergence of each clone from the orthologous mtDNA using uncorrected *p*-distance in MEGA 5 [58]. Finally, we calculated base composition (AT%) of each clone and tested whether the base compositions of numts were statistically homologous to the orthologous reference sequence using matched-pairs Bowker’s test for symmetry [59] as implemented in Seqvis [60].

### Phylogenetic analyses

To determine the pattern of nuclear insertion of mtDNA, we conducted a series of phylogenetic analyses by simultaneously analyzing numts and the orthologs. Specifically, we used two different taxon sampling strategies in order to address two separate phenomena. It is reported that nuclear insertion can happen multiple times within a species and that some numts can go through gene duplications [2], [49]. To explore this phenomenon in Orthoptera, we first created gene-specific and taxon-specific matrices, totaling 77 matrices (28 for COI, 25 for COII, and 24 for ND5). For each matrix, we included all of the numts generated from a given taxon as well as the orthologous mtDNA sequences of all ingroup and outgroup taxa. After each phylogenetic analysis, we examined the resulting topology and the relative placements of the numts to the orthologs to determine the patterns of nuclear integration. Hazkani-Covo [15] and Song et al. [18] reported that different taxa can share similar numts if the nuclear insertion of mtDNA occurred in the common ancestor before species divergence. Song et al. [18] named this type of numts as synaponumts. In order to test whether there were ancient synaponumts that were integrated in the nuclear genome of the common ancestors of different orthopteran families, we created three gene-specific matrices (COI, COII, ND5), containing numts of all taxa and the orthologs of all ingroup and outgroup taxa. If the synaponumts were present, we would recover a clade consisting of numts from different taxa, which would help us infer the relative timing of nuclear integration as well. For all analyses, we first aligned the nucleotide data in MUSCLE [61] using default parameters. Phylogenetic analyses were carried out in a maximum likelihood framework. We applied the GTRCAT model in RAxML 7.2.8 [62] on XSEDE (Extreme Science and Engineering Discovery Environment, https://www.xsede.org) through CIPRES Science Gateway [63]. Nodal support was evaluated using 1,000 replications of rapid bootstrapping implemented in RAxML. The resulting topologies were examined using Dendroscope (ver. 2.7.4) [64]. All matrices and resulting phylogenies have been deposited to TreeBase (Submission number 15850).

## Results

### Sequence characterization

**Figure 1:**
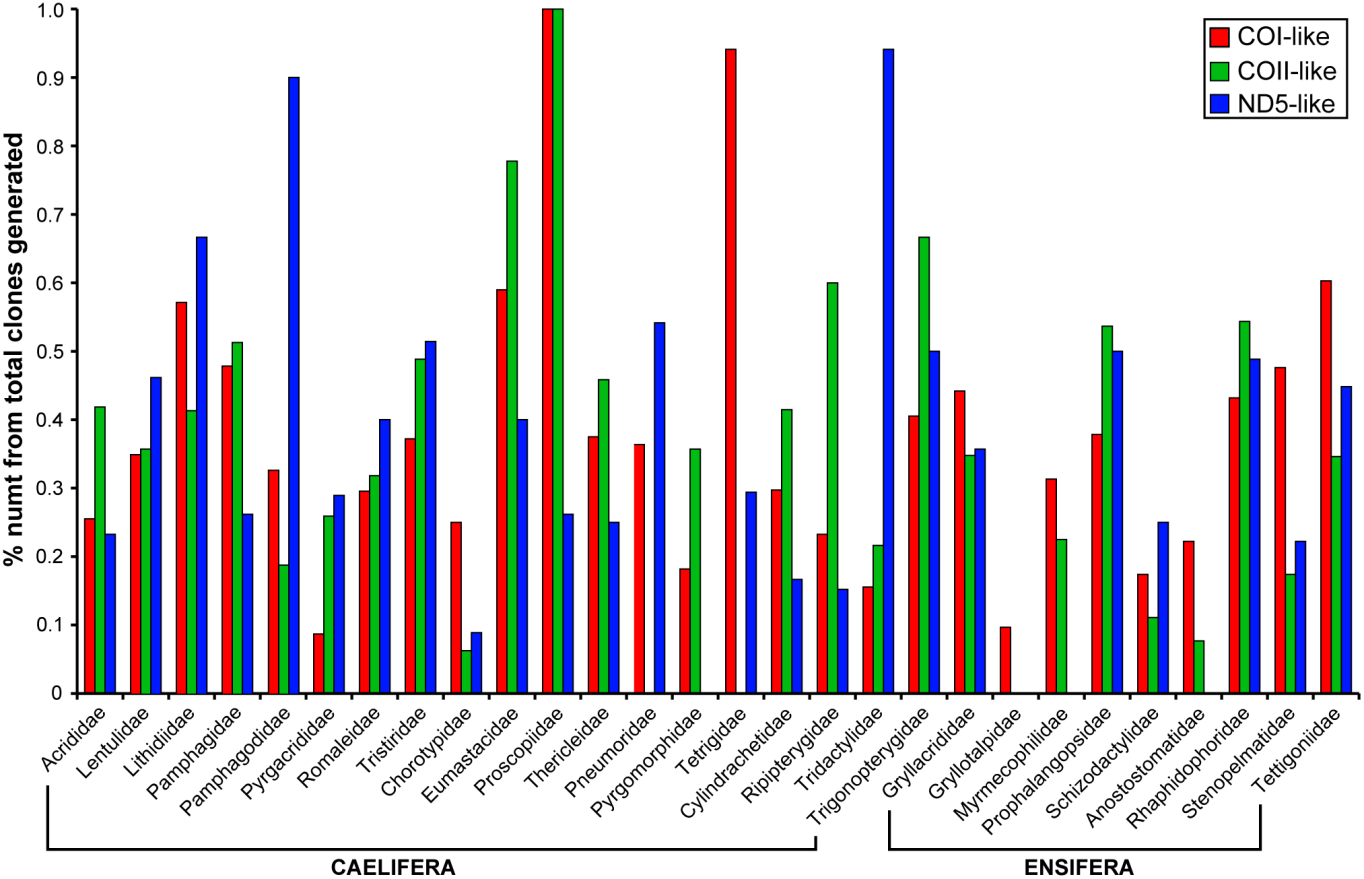
Numts of three mitochondrial genes (COI, COII, ND5) are extremely abundant across the phylogenetic diversity within Orthoptera. The y-axis shows the proportion of numts from the total number of clones generated based on PCR using conserved primers. If nearing 1, most clones generated are numts. If nearing 0, most clones generated are orthologous mtDNA.

We generated a total of 1,213 cloned sequences for COI gene from 28 orthopteran families, 817 for COII gene from 25 families, and 874 for ND5 gene from 24 families (Table 1). For COII and ND5 genes, we could not amplify and clone for three (Pneumoridae, Tetrigidae, Gryllotalpidae) and four families (Pyrgomorphidae, Myrmecophilidae, Gryllotalpidae, Anostostomatidae), respectively. The size of the resulting clones ranged from 60 to 1,882 bp (Table S3), which meant that the potential PCR error ranged from 0.009 to 0.2823 bp per product according to the error rate of the polymerase used [57]. Because even the highest possible error rate is lower than 1 bp per PCR, we considered the potential for false positives negligible. For all three mitochondrial genes, we recovered both the clones that were identical to the orthologous reference sequences and those that were uniquely different from the orthologs. One exception was found in Proscopiidae, in which none of the cloned sequences of COI and COII genes was identical to the orthologs, suggesting that numts were preferentially amplified. The proportion of the clones that were identical to the orthologs varied considerably across taxa and genes, but on average, only about 60% of the clones were identical to orthologs (60.55% for COI, 59.45% for COII, 59.67% for ND5). None of the taxa had 100% of the clones identical to the orthologs regardless of the genes, indicating that PCR always co-amplified a large number of mtDNA-like non-orthologous genes. Across the diversity of orthopteran lineages we sampled, we did not find a clear taxonomic bias in terms of the amount and the type of mtDNA-like sequences recovered. In some taxa, the prevalence of COI-like sequences was higher than the other genes, but in other taxa, COII-like or ND5-like sequences were more prevalent than the others (Fig. 1).

Because numts are known to accumulate random mutations [2], we characterized whether the cloned sequences contained premature stop codons, insertions, deletions or point mutations when compared with the orthologs (Table S3). We found that the proportion of the clones with stop codons or indels among the total number of numts generated for each gene per taxon was in general small (Table 1). The mean proportion was 18.94% for COI, 28.88% for COII, and 39.53% for ND5. Across all three genes, this proportion ranged from 0% (none of the numts having stop codons or indels) to 100% (all of the numts with stop codons or indels).

**Figure 2:**
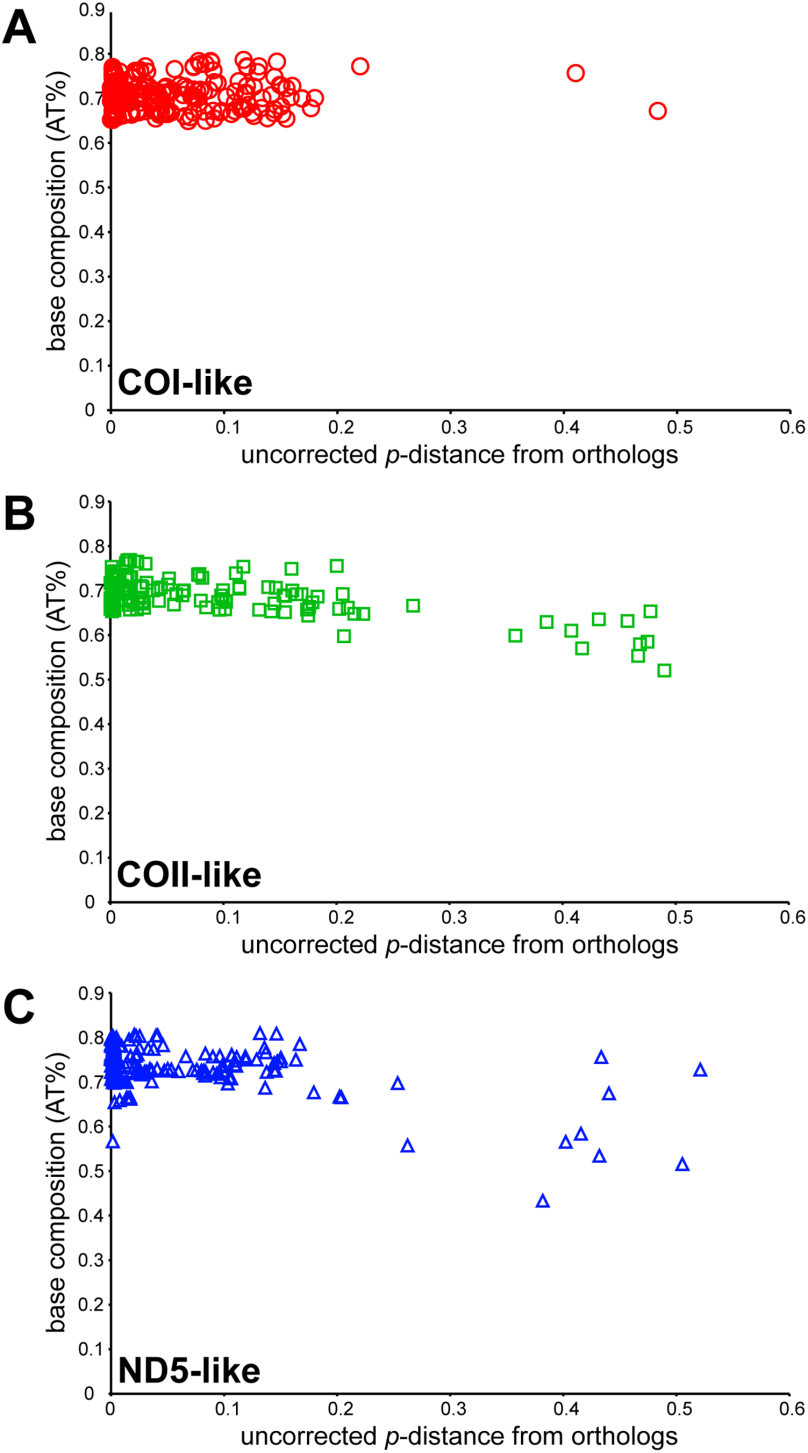
Characteristics of numts as measured by base composition and uncorrected p-distance from orthologs. A: COI-like numts; B: COII-like numts; C: ND5-like numts.

Because the base composition of mtDNA is inherently biased toward A and T [39], [72], we would expect numts to be less biased toward A and T, especially when they have been integrated into the nuclear genome for a long time [2]. Thus, we calculated base composition (AT%) of the numts and compared against the orthologs using matched-pairs Bowker’s test for symmetry [59]. We found that nearly all of the clones had statistically similar base compositions to the orthologous reference for all three genes across Orthoptera (Table S3). We found that a large number of numts in fact had very high sequence similarities to the orthologs. For example, more than half of all numts (259 COI-like, 185 COII-like, and 205 ND5-like numts) had less than 1% sequence divergence from the orthologs as calculated by uncorrected *p*-distance. As for the divergent numts, we found that only 3 out of 510 COI-like numts had statistically different base compositions (p<0.05) from COI gene, all of which were from Pneumoridae. Among COII-like numts, we found that 16 out of 324 clones had different base compositions, which were from Lentulidae (4), Lithidiidae (1), Pamphagidae (4), Prophalangopsidae (4), Tridactylidae (2), and Trigonopterygidae (1). For ND5-like numts, we found 8 out of 336 clones to have different base compositions, which were from Lentulidae (1), Lithidiidae (1), Pamphagodidae (2), Pneumoridae (1), Prophalangopsidae (2) and Trigonopterygidae (1). Most of these highly divergent numts, which also could be confirmed to have high uncorrected *p*-distances from the orthologs, had relatively lower AT% compared to the orthologs (Fig. 2), and this pattern was especially evident in COII-like and ND5-like numts.

### Phylogenetic distribution of numts

**Figure 3:**
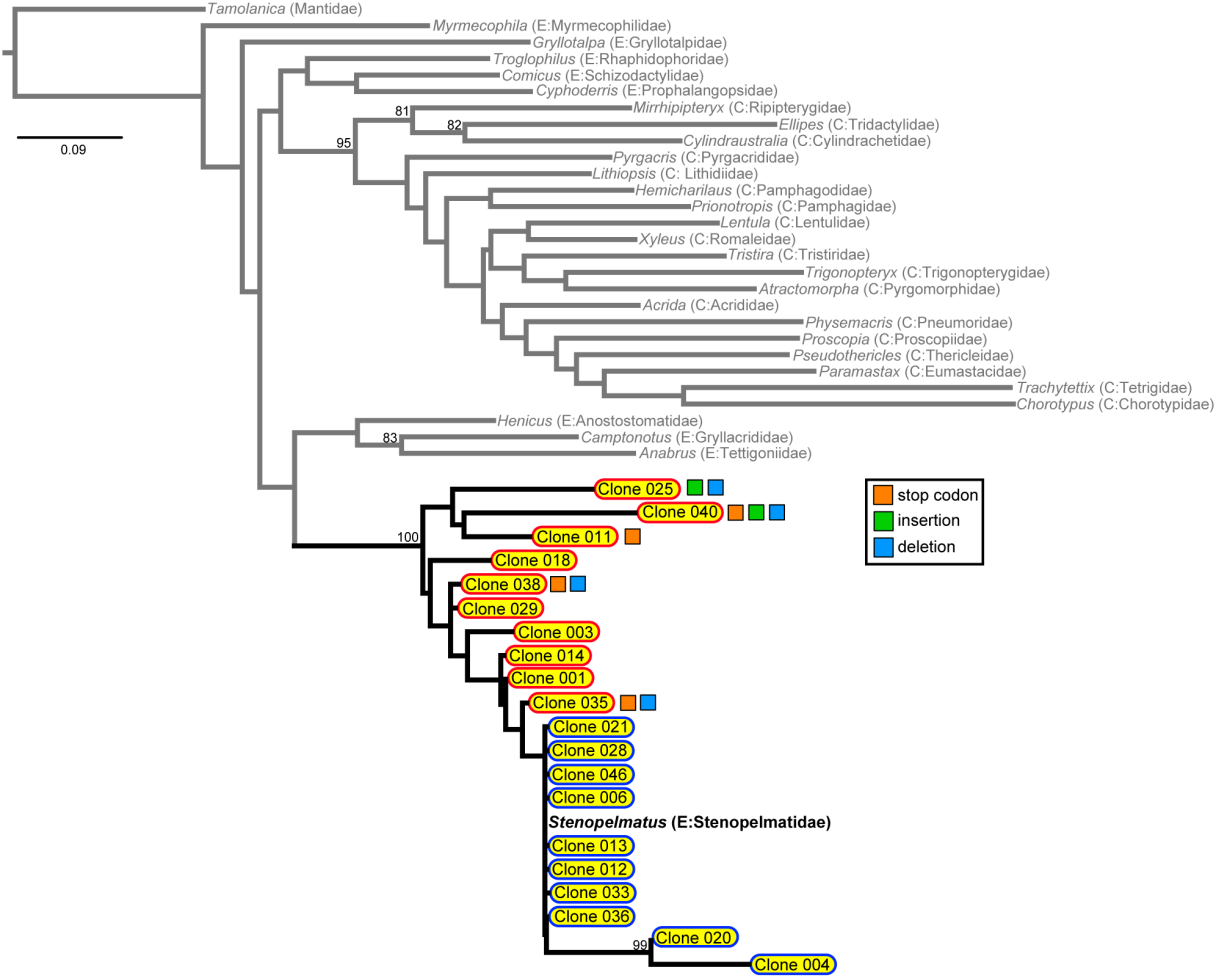
Co-analysis of numts and mtDNA using COI-like numts of *Stenopelmatus fuscus* (Ensifera: Stenopelmatidae). Grey terminals represent orthologous COI across Orthoptera. The maximum likelihood analysis recovered a monophyletic group consisting of COI-like numts and the ortholog from *S. fuscus*. Paleonumts are denoted by yellow with red outline, and neonumts are denoted by yellow with blue outline. Those numts with premature stop codons, insertions, and deletions, are indicated by orange, green, and blue squares, respectively. Numbers on nodes indicate bootstrap support values.

When the numts of any given orthopteran species were simultaneously analyzed with their orthologs in a phylogenetic framework, we recovered a very similar pattern across all of the 77 separate analyses (28 for COI, 25 for COII, and 24 for ND5), regardless of taxa or genes. To illustrate this point, we present a result from one such analysis (COI analysis for *Stenopelmatus fuscus*) (Fig. 3). Because the analysis was based on a small fragment of COI gene (Folmer region), which had insufficient phylogenetic information enough to resolve deep nodes across broad span of time, the resulting topology was incongruent with the currently accepted taxonomic classification for Orthoptera. However, we consider this point to be irrelevant because the objective of this particular analysis was to explore how numts would be placed relative to the respective orthologs. In this analysis, we recovered a strong clade consisting of COI-like numts and the orthologous COI of *Stenopelmatus* (Fig. 3). Within this clade, however, we recovered several subclades consisting only of numts, as well as one clade that included the ortholog and several numts with very short branch lengths. We were able to deduce the relative timing of nuclear integration based on the idea that the orthologous COI would represent extant, contemporary mtDNA. We then categorized the numts into two classes according to their phylogenetic placements relative to the ortholog as well as their branch lengths. The first type was the ancient numt or “paleonumt” which represented the nuclear insertion in the past before mtDNA took its current form. These paleonumts had characteristically longer branch lengths and did not closely group with the ortholog. In some case, these paleonumts would form a clade of their own, indicating either repeated nuclear insertion events in a short period of time in the past or a single nuclear insertion followed by gene duplication events [29], [31], [49], [73]. The paleonumts were often quite divergent from the ortholog in terms of *p*-distance and sometimes had stop codons and indels. The second type was the recent numt or “neonumt” which did not have enough time to accumulate many mutations and thus formed a polytomous clade with the ortholog. These neonumts were often characterized as having very short branch lengths and only a few base pair differences from the ortholog. However, some of these neonumts could be quite divergent from the ortholog, possibly if the particular region of nuclear genome that these numts were integrated happened to evolve rapidly. Among COI-like numts of *Stenopelmatus*, we found two such divergent neonumts. The neonumts are similar to the “cryptic numts” proposed by Bertheau et al. [33] in that they are both characterized by small differences from the orthologs, but the neonumts are conceptually more refined because the definition is explicitly based on their phylogenetic position relative to the orthologs.

We categorized the resulting numts of COI, COII, and ND5 across Orthoptera into paleonumts and neonumts according to the 77 separate phylogenetic analyses. In most cases, both types of numts were recovered regardless of the genes (Table 2). In some species, there were more neonumts than paleonumts, while in other species the opposite pattern was found. However, the prevailing pattern across Orthoptera was that there were more neonumts than the paleonumts (Table 2).

**Table 2:** All orthopteran families have two types of numts.

Taxonomic Information				COI-like		COII-like		ND5-like	
Suborder	Superfamily	Family	Species	paleonumt	neonumt	paleonumt	neonumt	paleonumt	neonumt
Caelifera	Acridoidea	Acrididae	*Acrida willemsei*	5	7	2	16	1	9
Caelifera	Acridoidea	Lentulidae	*Lentula callani*	3	12	7	8	0	12
Caelifera	Acridoidea	Lithidiidae	*Lithiopsis carinatus*	18	6	12	7	8	8
Caelifera	Acridoidea	Pamphagidae	*Prionotropis hystrix*	10	12	16	4	5	6
Caelifera	Acridoidea	Pamphagodidae	*Hemicharilaus monomorphus*	2	13	1	2	25	2
Caelifera	Acridoidea	Pyrgacrididae	*Pyrgacris descampsi*	0	2	5	9	1	10
Caelifera	Acridoidea	Romaleidae	*Xyleus modestus*	2	11	1	13	1	17
Caelifera	Acridoidea	Tristiridae	*Tristira magellanica*	6	10	6	15	11	7
Caelifera	Eumastacoidea	Chorotypidae	*Chorotypus fenestratus*	1	8	0	1	0	4
Caelifera	Eumastacoidea	Eumastacidae	*Paramastax nigra*	14	9	0	7	6	12
Caelifera	Eumastacoidea	Thericleidae	*Pseudothericles compressifrons*	5	7	2	9	1	9
Caelifera	Pneumoroidea	Pneumoridae	*Physemacris variolosa*	5	11	-	-	8	5
Caelifera	Proscopioidea	Proscopiidae	*Proscopia* sp.	8	0	0	8	1	10
Caelifera	Pyrgomorphoidea	Pyrgomorphidae	*Atractomorpha sinensis*	0	4	1	14	-	-
Caelifera	Tetrigoidea	Tetrigidae	*Trachytettix horridus*	13	19	-	-	0	5
Caelifera	Tridactyloidea	Cylindrachetidae	*Cylindraustralia* sp.	4	7	1	16	2	4
Caelifera	Tridactyloidea	Ripipterygidae	*Mirrhipipteryx andensis*	6	4	1	2	0	7
Caelifera	Tridactyloidea	Tridactylidae	*Ellipes minuta*	1	6	4	4	15	1
Caelifera	Trigonopterygoidea	Trigonopterygidae	*Trigonopteryx* *hopei*	9	6	3	1	3	13
Ensifera	Gryllacridoidea	Gryllacrididae	*Camptonotus* *carolinensis*	2	17	2	14	3	12
Ensifera	Grylloidea	Gryllotalpidae	*Gryllotalpa pluvialis*	0	3	-	-	-	-
Ensifera	Grylloidea	Myrmecophilidae	*Myrmecophila* *manni*	1	25	4	5	-	-
Ensifera	Hagloidea	Prophalangopsidae	*Cyphoderris monstrosa*	4	10	14	8	3	18
Ensifera	Schizodactyloidea	Schizodactylidae	*Comicus campestris*	1	7	0	1	1	10
Ensifera	Stenopelmatoidea	Anostostomatidae	*Henicus brevimucronatus*	0	4	0	1	-	-
Ensifera	Stenopelmatoidea	Rhaphidophoridae	*Troglophilus neglectus*	13	6	10	15	15	6
Ensifera	Stenopelmatoidea	Stenopelmatidae	*Stenopelmatus fuscus*	10	10	1	7	0	6
Ensifera	Tettigonioidea	Tettigoniidae	*Anabrus simplex*	27	14	2	7	1	12
			**Total**	**170**	**250**	**95**	**194**	**111**	**205**

The first type is the ancient numt or “paleonumt” which represents the nuclear insertion in the past before mtDNA took its current form. The second type is the recent numt or “neonumt” which did not have enough time to accumulate many mutations.

Because the paleonumts potentially represented fossilized mtDNA lodged in the nuclear genome [13], [40], we explored how ancient these paleonumts would be by phylogenetically analyzing numts from all taxa simultaneously. If the numts from two divergent taxa formed a clade, this would be a strong indication that those particular numts were inserted into the nuclear genome of the most recent common ancestor (MRCA) of those two taxa [15], [18]. Among COI-like numts, we found one clade, which consisted of a numt from *Paramastax* (Eumastacidae) and a numt from *Pyrgacris* (Pyrgacrididae). Interestingly, these numts did not differ much from the orthologs in terms of base compositional bias, but were quite divergent in terms of *p*-distance (Table 3). Among COII-like numts, we found two clades, one of which consisted of numts from *Lentula* (Lentulidae), *Prionotropis* (Pamphagidae), and *Ellipes* (Tridactylidae), and another clade also consisting of numts from *Lentula* and *Prionotropis*. Among ND5-like numts, we found one clade consisting of two divergent numts from *Hemicharilaus* (Pamphagodidae), one numt from *Lentula* (Lentulidae), one numt from *Physemacris* (Pneumoridae), and two numts from *Cyphoderris* (Prophalangopsidae). Between COII-like and ND5-like numts, some had considerably different base compositional bias from the orthologs, while some had similar AT% as the orthologs. In all cases, these paleonumts were highly divergent from the orthologs in terms of sequence divergence (Table 3). In order to determine a plausible timing of the nuclear insertion for these paleonumts, we performed a literature search to find records for the oldest definitive fossils for MRCA for each clade [74]–[77], which is presented in Table 3. We determined that the oldest possible numts were ND5-like numts, which dated back at least to the Jurassic period (182–201 MYA).

**Table 3:** Examples of clades formed by paleonumts from different orthopteran species.

Numt type	Family	Numt name	Ortholog AT%	NumtAT%	p-distance from ortholog	Age of MRCAbased on fossil data
COI-like numts	Eumastacidae	OR407_C039	0.676	0.678	0.410	145–163.5 MYA [77]
	Pyrgacrididae	OR317_C179	0.603	0.602	0.483	(Oldest Eumastacidae)
COII-like numts	Lentulidae	OR295_C020	0.742	0.521	0.490	98.7–108 MYA [75]
	Pamphagidae	OR151_C053	0.693	0.570	0.417	(Oldest Tridactylidae)
	Tridactylidae	OR153_C069	0.655	0.653	0.478	
COII-like numts	Lentulidae	OR295_C011	0.742	0.636	0.432	33.9–38 MYA [76]
	Pamphagidae	OR151_C069	0.693	0.629	0.386	(Oldest Acridoidea)
ND5-like numts	Lentulidae	OR295_C207	0.781	0.756	0.434	182–201 MYA [74]
	Pamphagodidae	OR540_C135	0.725	0.728	0.521	(Oldest Prophalangopsidae)
	Pamphagodidae	OR540_C121	0.725	0.516	0.505	
	Pneumoridae	OR293_C021	0.757	0.674	0.440	
	Prophalangopsidae	OR021_C021	0.709	0.584	0.416	
	Prophalangopsidae	OR021_C022	0.709	0.565	0.402	

The recovery of these clades indicates that the nuclear insertion event probably occurred in the most recent common ancestor (MRCA) of the species forming the clades. Numt name indicates the specific cloned sequence number used in the study, available in Table S3. Ortholog AT% indicates the base composition of the orthologous mtDNA sequence of the corresponding numt. Numt AT% is the base composition of the specific numts below to show how similar or different they are from the ortholog. *p*-distance from ortholog indicates the uncorrected p-distance of the numt sequence from the corresponding ortholog. In general, these paleonumts are highly divergent from the orthologs. Age of MRCA based on fossil data is determined from the oldest known fossil for particular clades, thus showing the maximum date of nuclear insertion.

## Discussion

### Numts of multiple mitochondrial genes are rampant in all orthopteran lineages

One of the first reports that demonstrated the existence of mtDNA-like sequences in the nuclear genome was based on a study of an orthopteran insect, *Locusta migratoria* [4], and since then, Orthoptera has become a model invertebrate system for studying numts. Zhang and Hewitt [52] discovered the presence of a highly conserved mitochondrial control region in the nuclear genome of *Schistocerca gregaria*, and Bensasson et al. [49] reported ND5-like numts from several grasshopper species in Melanoplinae [Podisminae], Calliptaminae, Gomphocerinae, and Cyrtacanthacridinae. Song et al. [24] showed that conventional PCR using Folmer primers could coamplify COI-like numts in four subfamilies of grasshoppers, which might overestimate the number of species under DNA barcoding method. Moulton et al. [23] documented the presence of COI-like numts in 10 different orthopteran families. Most recently, Song et al. [18] showed the prevalence of COI-like numts in 21 species of *Schistocerca*, suggesting that closely related species in lower-level taxonomic groups could have high accumulation of numts. Our present study represents a bold attempt to comprehensively document the presence of numts across the major lineages of Orthoptera.

In this study, we show that numts of multiple mitochondrial genes (COI, COII, and ND5) are extremely prevalent in every single family examined, representing members of all 14 known superfamilies across Orthoptera. An earlier study suggested that the prevalence of numts might be lineage-specific [24], and the present study provides an excellent opportunity to test whether different orthopteran lineages vary in the amount of numts that they harbor. By comparing the proportion of numts from the total number of clones generated per species per gene (Fig. 1), we clearly show family-level variations in the amount of numts for any given gene, but there does not appear to be a consistent pattern across genes. For example, a taxon with a high proportion of COI-like numts does not necessarily have high proportions of COII-like or ND5-like numts. More frequent is a pattern where a taxon has a relatively high amount of one particular type of numts compared to other two types. Furthermore, there is no discernable and consistent pattern of numt accumulation between different superfamilies or different suborders. This pattern suggests that, at least within Orthoptera, the presence of a large amount of numts is a norm, rather than an exception.

It is important to consider that processes other than the nuclear insertion of mtDNA can also result in coamplification of mtDNA-like sequences [23]. Microheteroplasmy due to somatic mutation [65], [66], divergent heteroplasmy due to biparental inheritance or paternal leakage [67]–[70], or nuclear insertion of heteroplasmy [18] can potentially generate mtDNA-like sequences using the methods we used in this study. A recent study focusing on human mitochondrial RNA demonstrated a remarkably high level of intraspecific sequence variation suggesting a high level of heteroplasmy [71]. However, it is very difficult to distinguish between numts and heteroplasmies with confidence in PCR-based studies. Moulton et al. [23] and Song et al. [18] used sequence divergence of the amino acid sequences as a criterion to define heteroplasmies, but this is an arbitrary definition and there is a possibility that some of the sequences they defined as heteroplasmies might actually be numts. Therefore, in this study we considered all resulting mtDNA-like sequences as numts with a caveat that a small portion of sequences that appear to be functional might be possible heteroplasmies.

A typical metazoan mitochondrial genome consists of 37 genes (13 protein-coding, 2 ribosomal RNA, and 22 tRNA genes) [72], but it has not been clear whether certain genes are more prone to be inserted into the nuclear genome than others [21]. In this study, we have deliberately selected three protein-coding genes that are different in several characteristics. COI and COII are physically close to each other and encoded on the major strand, while ND5 is about 5,000 bp away from COII and encoded on the minor strand [39], [72]. Cytochrome *c* oxidases are involved in the respiratory chain that catalyzes the reduction of oxygen to water and NADH dehydrogenase are involved in forming a large enzyme complex known as complex I, which is important for oxidative phosphorylation [72]. Therefore, if the nuclear insertion of mtDNA were not random, it would be possible to observe gene-specific differences in abundance of numt accumulation. In fact, Tsuji et al. [21] showed that, in mammals, numts originated from D-loop (control region) of the mitochondrial genome were underrepresented among all the identifiable numts, suggesting that the pattern of numt insertion might not be random (but see Soto-Calderón et al. [19]). In our study, we do not find any evidence that a certain mitochondrial gene is more prone to be inserted into the nucleus because we find that on average about 40% of the clones of PCR amplicons are different from the orthologous sequences regardless of the genes. In other words, all three mitochondrial genes have been similarly inserted into the nucleus. Certainly, it is difficult to generalize this pattern across the entire mitochondrial genome, but we strongly suspect that at least for the coding region, the numt insertion is random. This finding is congruent with a pattern found in humans [19], [78]. Previous surveys using the BLAST search of mitochondrial genome against the nuclear genome [6], [16], [21], [42], [79]–[81] seem to suggest that the nuclear insertion of mtDNA occurs based on fragments of mtDNA, which may or may not include a specific gene in its entirety. There is also evidence for direct transfer of mtDNA to the nucleus that does not involve a cDNA intermediate [5], [82]. Thus, it appears that nuclear insertion of mtDNA is a random event, and it is reasonable to suspect that numts of all 37 mitochondrial genes can be found in many different lineages of Orthoptera.

One caveat in our study is that our numt generation method relied heavily on the efficiency of primers to coamplify numts. By design, a PCR-based method can only recover numts that have high sequence similarities at the primer binding sites. Also, we only generated about 50 clones per sample and it is likely that more clones would result in a more complete sampling of extant numts. Therefore, the amount of numts reported here would be only a subset of the total numt diversity in the nucleus. This demonstrates that there may be a vast amount of numt diversity waiting to be discovered in Orthoptera. Such diversity can be explored further in depth in the future using next-generation sequencing approaches, which will allow characterizing all of the numts lodged in the nuclear genome without the limitation of the primer binding sites.

### Nuclear insertion of mtDNA has occurred continuously throughout the diversification of Orthoptera

We find that the nuclear insertion of mtDNA must have occurred continuously and very frequently throughout the diversification of Orthoptera. The ongoing numt insertion has been reported from humans [81], [83] as well as other eukaryotes [2], [5], and our findings are congruent with the reported patterns. In this study, we have broadly categorized numts into two different types based on their phylogenetic placements relative to the orthologs and their branch lengths: paleonumts and neonumts. Both types are clearly present among the numts of all three genes and we find more neonumts than the paleonumts (Table 2). This continuous pattern of numt insertion indicates that the nuclear genome can be thought of as a natural repository for mtDNA mutations throughout the organism’s evolutionary history.

The prevalence of neonumts, representing the nuclear insertion of contemporary mtDNA, has been demonstrated consistently in previous studies [14], [18], [28], [30], [33], [43], [49], [73], [84], [85] and our findings bolster the idea that this must be an ongoing process. Several mechanisms of numt insertion have been proposed (see Hazkani-Covo et al. [5] for review), although it is not clear if one particular mechanism is more prevalent than the others. It may be possible that multiple mechanisms have contributed to the diversity and abundance of numts in Orthoptera. Regardless of the mechanisms, the nuclear insertion of mtDNA is a physiological process that must occur within an individual and the numts that are transmitted across generations must have been inserted during gametogenesis. Unlike mtDNA, which is maternally inherited [72], numts must be inherited both paternally and maternally. If the rate of nuclear insertion were naturally high for a given organism, which seems to be the case for Orthoptera, the rate of numt transmission across generations would also be very high. In such a scenario, an individual will harbor numts that have originated both paternally and maternally and if this idea can be extrapolated further, a given individual must harbor numts that are representative of its population, as well as of a species as a whole in its nuclear genome.

Numts are considered molecular fossils of mtDNA [13], [40], which implies that once in the nucleus, their mutation rate would slow down relative to the natural mutation rate of mtDNA [3]. The rate of numt mutation certainly depends on the insertion site [29], but the published reports seem to suggest that the integrity of mtDNA-likeness is often well preserved, implying a generally slower rate of numt mutation. In fact, paleonumts that are highly divergent from the contemporary mtDNA have been discovered in numerous taxa [2], [5], [44], and Hazkani-Covo [15] discovered similar numts in the genomes of human, chimpanzee and orangutan, that must have been inserted at least 13 million years ago in the common ancestor of the three modern primates. The oldest numts reported from human is inferred to be at least 58 million years old [80]. The presence of these paleonumts suggests that numts can potentially remain intact for a long time. However, it is unclear how long can numts stay intact in the nucleus before they mutate so much as to become indistinguishable from the rest of nuclear genome. Our large taxon sampling across the phylogeny of Orthoptera allows addressing this question because we have discovered some paleonumts shared by highly divergent families. By phylogenetically analyzing numts from multiple taxa simultaneously, we have discovered clades that consist of numts from different families, suggesting that these are synaponumts (shared derived numts), which represent nuclear insertion in the common ancestor, which persist in the nuclear genome of descendant species [18]. Often these numts are quite divergent from the orthologs as well as from each other, and when they do form a clade, the terminal branches are characteristically long, and the nodal support values are relatively low. Therefore, it is difficult to be confident if the resulting clades represent accurate relationships or an analytic error such as long-branch attraction, which may occur even in a maximum likelihood framework [86]. Nevertheless, if these relationships are real, then we can make some interesting inferences. It is challenging to directly estimate the time of nuclear insertion based on sequence characteristics alone because there is not a solid model for calculating past mutation rate in the nuclear genome relative to the mitochondrial genome (but see Thalmann et al. [43]). However, in the case of Orthoptera, there are numerous fossils available to indirectly estimate the oldest date of nuclear insertion (Table 3). For example, we have recovered a strong clade consisting of COI-like numts from Eumastacidae and Pyrgacrididae supported by a bootstrap value of 100. This relationship is very robust despite the fact that two sequences are divergent from each other with the uncorrected *p*-distance of 0.293 and 145 point mutations (∼22% sequence differences). Pyrgacrididae (*Pyrgacris descampsi*) is an obscure grasshopper family endemic to Reunion Island in the Indian Ocean [87], [88]. Eumastacidae (*Paramastax nigra*) is a family primarily found in the tropics [89], and the particular species used in our study occurs in Peru. Two families belong to different superfamilies, Pyrgacrididae in Acridoidea and Eumastacidae in Eumastacoidea and they are morphologically highly divergent from each other. Eumastacidae is older than Pyrgacrididae, and the oldest definitive eumastacid fossil is known from the Jurassic (145–163.5 MYA) [90]. Therefore, we can deduce that the nuclear insertion event must have occurred in the common ancestor between these two families, which must be at least 150 million years ago, which implies that numts can persist in the nuclear genome for a very long time. The oldest numts we can infer from our study appear to have been inserted in the common ancestor among Prophalangopsidae, Pneumoridae, Lentulidae and Pamphagodidae, which probably occurred in the Jurassic Period. What is the most surprising is the fact that we were able to coamplify these paleonumts using conventional PCR primers, which indicates that the primer binding sites of these numts have remained intact for such a long period of time.

### Why so many numts in Orthoptera?

It is clear that there is a large amount of diverse classes of numts in Orthoptera. However, it is likely that mechanisms in addition to direct nuclear insertion are responsible for this diversity. Once integrated into the nuclear genome, numts are subject to molecular processes such as duplication, transposition, and deletion [2], [5]. Of these, duplication has been implicated as a main process for the large amount of numts in several divergent taxa [31], [73], [80], [91]. When numts are duplicated in the nucleus, a phylogenetic analysis can recover the duplicated numts as a monophyletic group consisting only of themselves [2]. In fact, this is an extremely prevalent pattern in our study, found across many taxa regardless of the genes.

Why are there so many numts in Orthoptera? Among insects, Orthoptera is reported to have the largest genome size [47], [92], which ranges from 1.52 to 16.56 Gb [48]. Taxonomically, Acrididae has the largest genome (3.76–16.56 Gb), followed by Gryllacrididae (9.34 Gb), Gryllotalpidae (8.18 Gb), Tettigoniidae (2.59–7.75 Gb), Eumastacidae (3.67–3.91 Gb), and Tridactylidae (2.58 Gb) [47], [48]. Gryllidae has the smallest genome within Orthoptera (1.52–2.62 Gb), which is still ten times larger than the genome of *Drosophila melanogaster* [48]. Indeed, there seems to be a strong positive correlation between the genome size and the prevalence of numts across animals, plants, fungi, and protists [2], [5], suggesting that a large genome size allows for an increased probability for numts to be inserted into the nuclear genome. Bensasson et al. [46] documented that the rate of DNA loss due to deletion, which is crucial for keeping the nuclear genome compact and efficient, is much slower in the brown mountain grasshopper (*Podisma pedestris*) relative to *Drosophila* or the cricket *Laupala*, which may contribute to genomic gigantism. Thus, even if the rate of nuclear insertion may be relatively uniform across species, the slow rate of DNA loss in Orthoptera would result in a relatively high rate of numt accumulation [46], [93]. A recently sequenced genome of the *Locusta migratoria* is 6.5 Gb in size [92], and 60% of the assembled genome reportedly consists of repetitive elements, including DNA transposons and LINE retrotransposons, which contribute to the large genome size. The abundance of retrotransposons in *L. migratoria* is particularly intriguing, which might be a general pattern across Orthoptera. Based on a genomic survey of primate numts, which showed that these numts tended to insert near retrotransposons, Tsuji et al. [21] proposed a hypothesis that the activity of retrotransposons may be related to frequent numt insertions. Therefore, the large genome size, the slow rate of DNA loss, and the abundance of retrotransposons that can potentially insert numts directly to the nuclear genome might have collectively contributed to the extremely large amount of numts in Orthoptera.

### Concluding remarks

Numts have been called “molecular fossils in the nucleus” [13], [40], “evolution’s misplaced witnesses” [2] and “molecular poltergeists” [5], and what we know today is that numts are extremely widespread across eukaryotes [2], [5], [6]. Based on the most recent survey of numts using completely sequenced genomes, Hazkani-Covo et al. [5] reported only 8 species out of 85 eukaryotes had no numts detected from their genomes. As more genomes become available through next generation sequencing technologies, we will have a better understanding of the extent and distribution of numts. It is probable that the presence of numts in eukaryotes is a norm, rather than an exception. In light of what we know about numts now, we can re-characterize the nature of numts. Unlike regular fossils, which are often rare, numts as molecular fossils are abundant and easy to find. Numts are the main witnesses of the past evolutionary events that affect mtDNA, and they are not as elusive as poltergeists any more, especially with the advances in sequencing technologies. Although numts have been often considered nuisances for molecular systematics [10], [22], [24], [26], [30], [33], [41], [50], [94], they have the potential to illuminate evolutionary history. The non-coding region of the nuclear genome, which is where numts are presumably inserted [49], can be thought of as a computer hard drive, which has saved numerous past versions of mtDNA, which are retrievable. With a careful investigation of these numts, we will be able to gain novel insights into the forgotten evolutionary history of organisms, which may not be directly accessible through available phylogenetic markers.

## Supporting Information

Table S1 Taxonomic information, collecting information, and voucher information for the taxa included in this study.
(XLSX)

Table S2 Specific primers developed and used in this study.
(XLSX)

Table S3 Detailed information about the clones generated for this study and their sequence characteristics in comparison with reference mitochondrial genes.
(XLSX)
